# Dynamic ESPI Evaluation of Deformation and Fracture Mechanism of 7075 Aluminum Alloy

**DOI:** 10.3390/ma14061530

**Published:** 2021-03-20

**Authors:** Shun Takahashi, Sanichiro Yoshida, Tomohiro Sasaki, Tyler Hughes

**Affiliations:** 1 Graduate School of Science and Technology, Niigata University, Niigata 9502181, Japan; f19b107f@mail.cc.niigata-u.ac.jp; 2 Department of Chemistry and Physics, Southeastern Louisiana University, Hammond, LA 70402, USA; sanichiro.yoshida@selu.edu (S.Y.); Joshua.hughes-2@selu.edu (T.H.)

**Keywords:** field theory, deformation and fracture, 7075 aluminum alloy, electronic speckle pattern interferometry

## Abstract

The deformation and fracture mechanism in 7075 aluminum alloy is discussed based on a field theoretical approach. A pair of peak-aged and overaged plate specimens are prepared under the respective precipitation conditions, and their plastic deformation behaviors are visualized with two-dimensional electronic speckle pattern interferometry (ESPI). The in-plane velocity field caused by monotonic tensile loading is monitored continuously via the contour analysis method of ESPI. In the plastic regime, the peak-aged specimen exhibits a macroscopically uniform deformation behavior, while the annealed specimen exhibits non-uniform deformation characterized by a localized shear band. The occurrence of the shear band is explained by the transition of the material’s elastic resistive mechanism from the longitudinal force dominant to shear force dominant mode. The shear force is interpreted as the frictional force that drives mobile dislocations along the shear band. The dynamic behavior of the shear band is explained as representing the motion of a solitary wave. The observed decrease in the solitary wave’s velocity is accounted for by the change in the acoustic impedance with the advancement of plastic deformation.

## 1. Introduction

Micro-fractures in structural materials can rapidly grow in their scale and directly lead to serious accidents. Even in aluminum alloys, known to be highly ductile materials, failure occurs in an uncontrollable fashion. Because of their high specific strength, corrosion resistance, weldability, and inexpensiveness, aluminum alloys are widely used for the parts of various structures. Thus, understanding of the fracture mechanism in aluminum alloys has been subjects of intensive study for a long time. However, much remains unexplained.

A challenging aspect in this regard is the difficulty in the description of the transition from plastic deformation to fracture. It is generally known that the fracture of ductile metals is initiated by non-uniformity in the microstructure, such as anisotropy of crystal grains, inhomogeneity of precipitates and solid solutions and develop to macroscopic non-uniformity. It is also known that the progression of localized yielding and plastic deformation develop to necking and eventually lead to fracture. However, the dynamics of the transitional stage is not well understood.

As an indicator of the progression from the micro- to macro-scale non-uniformity in deformation, the phenomenon known as the Portevin-Le Chatelier (PLC) effect [1] is of great interest. The PLC effect is typically observed in tensile experiments of aluminum alloy specimens and exhibits the following two characteristics. The first is the zigzag characteristics of the stress–strain curve known as the serration. The second is the local shear deformation called the “shear band”. The former represents the instability in the shear stress and the latter the local strain concentration associated with the stress instability. The most intriguing aspect of this effect is that it qualitatively relates the micro- and macro-dynamics of deformation and its progression to fracture. In the microscopic view, it is generally accepted that the origin of plastic instability is dynamic strain aging responsible for the dynamic interactions between mobile dislocations and solute atoms in alloys [2]. The effect of strain rate and the microstructural factors such as the amount of solute atom and grain size still has been theoretically, as well as experimentally investigated by a considerable number of researchers e.g., [3].

In order to connect those microscopic and macroscopic dynamics, and thereby deformation and fracture mechanisms quantitatively, it is essential to take a comprehensive approach that describes both microscopic deformation mechanisms, such as interatomic bonding and dislocation motion in crystals, and fracture mechanics, such as necking and cracking on the same theoretical foundation. A recent field theory of deformation and fracture [4,5] has the ability to meet the requirement. Based on the fundamental physical principle known as symmetry in physics, this theory describes all stages of deformation including elasticity, plasticity and fracture mechanism on the same basis without relying on phenomenology and scale-independently. According to this theory, deformation can be comprehensively described as the wave dynamics that govern the displacement field of deforming objects and the transition to fracture can be characterized by the change in the form of the wave. More specifically, as the deformation transitions from the deformation to fracturing stage, the wave changes its form from sinusoidal type waves to a solitary waves.

On the experimental end, a number of authors use various optical techniques to visualize the above-mentioned phenomena. Hassan et al. [6] presented the first comprehensive literature review on remote deformation measurement in the presence of discontinuities using the digital image correlation technique. Ranc et.al [7] use infrared pyrometry to visualize the heat generation during the shear band formation. These studies have correlated the temporal and spatial behavior of the shear band with the nature of serrations. However, they do not clarify the detailed mechanism underlying in the transition from deformation to fracture.

From the viewpoint of implementation of the above-mentioned field theory in a diagnostic algorithm, the optical interferometric technique known as the electronic speckle-pattern interferometry (ESPI) is useful. Yoshida et al. [8] discusses the specific wave types that differentiate stages of deformation in association with the corresponding optical interferometric fringe patterns. In [9], Yoshida et al. extend the discussion to the transition to the fracture and identify the shear band as manifestation of the solitary wave dynamics in association with micro-fracture and corresponding dislocation dynamics.

While ESPI is a powerful tool to implement the field theory, it is necessary to analyze a large number of interferometric fringe patters for diagnosis. It is desirable to use a computerized method to automate the fringe analysis, at least partially. Due to the non-uniformity nature of the deformation, the interference fringes are non-uniform spatially. In addition, the deformation in the late stage of plastic regime does not transient at a constant rate. These situations make it difficult to apply the conventional methods such as the phase shift method [10]. Recently, we have devised an algorithm to facilitate the process of fringe analysis under the given situation. Thus, the aim of this paper is twofold. In the first part, we present the computerized fringe analysis method. In the rest of the paper, we report our recent study in which we used this fringe analysis algorithm to visualize the non-uniform deformation and fracturing behavior in Al–Zn–Mg–Cu alloy specimens. We will discuss the physics behind the observed deformation behaviors and clarify the interconnection to the dislocation dynamics. The effect of heat treatment on the specimen is discussed as well.

## 2. ESPI Optical Measurement System and Analysis Algorithm

### 2.1. Optical Arrangement of Electronic Speckle Pattern Interferometry

**Figure 1:**
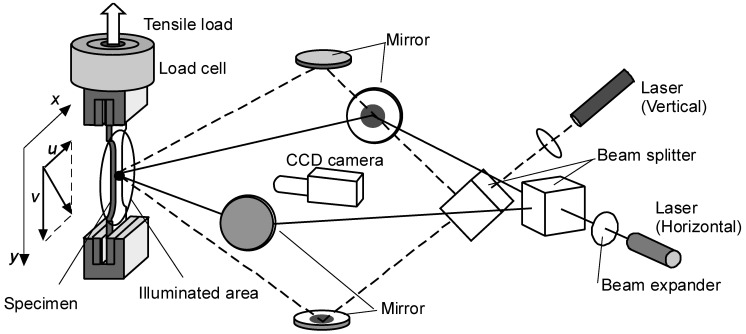
Optical setup of 2-D electronic speckle pattern interferometer [9].

The deformation behaviors of Al–Zn–Mg–Cu alloys were measured with the two-dimensional ESPI described in the previous work [9]. The experimental set-up is shown in Figure 1. The two-dimensional displacement fields in the directions *x* and *y* on the measured surface can be obtained by computing the intensity difference as a contour pattern, called the “fringe pattern”. The relation of the displacement ξ and the phase difference between the interferometric arms Δϕ is expressed by (see Appendix A):

(1) $$\Delta \phi =4\pi \frac{\xi \sin\theta }{\lambda }$$

In this study, the tensile test was conducted at a constant strain rate of 8.33 × 10^−3^ [s^−1^]. The fringe pattern was calculated for several time intervals, in order to maintain the accuracy of the analysis. The velocity v=∂ξ/∂t was calculated by dividing the displacement value by the time interval. The tensile load was measured with a strain gauge type load cell. The tensile strain was calculated from the displacement of crosshead and the gauge length of specimen.

### 2.2. Fringe Pattern Analysis

The displacement field can be obtained by extracting the fringe pattern. The intensity of superimposed speckles on the measurement point (*x*, *y*) is generally expressed by the following equation [11]:

(2) $$I(x,y)=2A^{2}+2A^{2}\cos\phi (x,y)$$

where, *A* is amplitude of light source, ϕ is the initial phase difference between the speckles resulting from the two interferometric arms. Since each speckle has phase, the phase ϕ can be approximated as taking a random distribution in the measurement field like white noise. The superimposed speckle intensities before and after the deformation are expressed as follows:

(3) $$I_{Before}(x,y)=2A^{2}+2A^{2}\cos\phi$$

(4) $$I_{After}(x,y)=2A^{2}+2A^{2}\cos(\phi +\Delta \phi )$$

where, Δϕ is the phase difference resulting from the deformation of material. The Δϕ can be estimated through the subtraction of the speckle intensities before and after. The absolute difference of intensity before and after deformation is calculated as follows:

(5) $$\left|I_{After}-I_{Before}\right|=4A^{2}\left|\sin\frac{2\phi +\Delta \phi }{2}\sin\frac{\Delta \phi }{2}\right|$$

**Figure 2:**
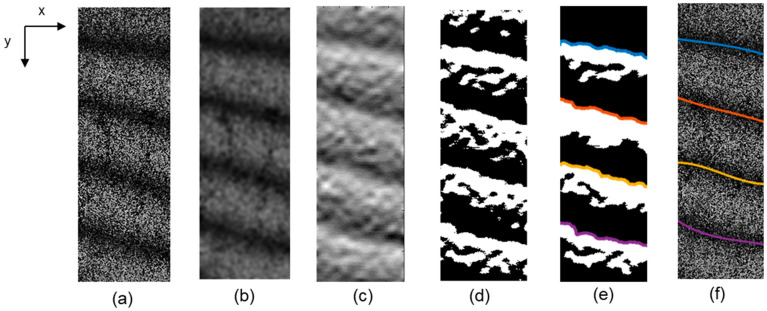
(**a**) original image (**b**) Gaussian filtered image (**c**) partial differential image (**d**) binarized image (**e**) morphology processed image (**f**) detected fringe line.

Here, the first term of right hand $\sin\frac{2\phi +\Delta \phi }{2}$ contains the initial phase of the superimposed speckle, while ϕ randomly changes depending on the material surface. Since the second term Δϕ/2 becomes zero when Δϕ is equal to a multiple of 2π regardless the ϕ, we can determine the phase Δϕ where the speckle appears as “dark fringes”. To determine the Δϕ reducing the speckle noise caused by the first term, image processing was conducted. The process consists of five steps of (i) speckle noise reduction using Gaussian filter, (ii) phase determination with partial differential image, (iii) binarization, (iv) morphological processing and detection fringes and (v) interpolation as shown in Figure 2. Details of the steps are described as follows:

- Step (i): Speckle noise reduction using Gaussian filter.

**Figure 3:**
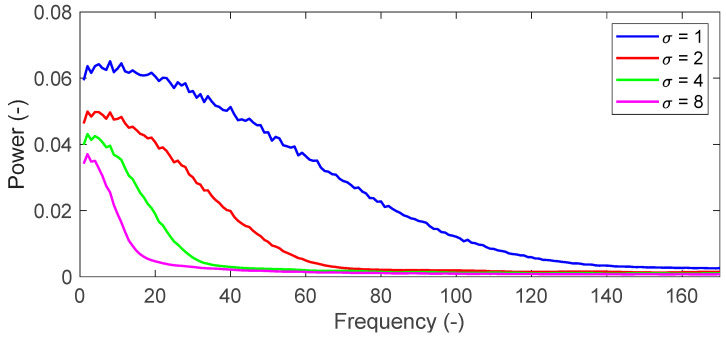
Frequency characteristics of the Gaussian filter.

Figure 2a shows a specific example of fringe pattern obtained in the tensile test of aluminum alloy. The fringe pattern represents in-plane displacement component *v*, in the direction *y*. The pattern includes four fringes due to the displacement on the measurement surface and the noise resulting from the speckle noise. To remove the high frequency component of noise, we applied a weighted Gaussian filter as follows:

(6) $$I_{filtered}(x,y)=\sum_{i}\sum_{j}h(i,j)I(i-x,j-y)$$

(7) $$h_{g}(i,j)=e^{-\frac{i^{2}+j^{2}}{2\sigma^{2}}}$$

(8) $$h(i,j)=\frac{h_{g}(i,j)}{\sum_{i}\sum_{j}h_{g}(i,j)}$$

where, *σ* is standard deviation of the Gaussian distribution, indexes *i* and *j* represent distances from center position of filter. The filtered image is obtained by a convolution between the intensity of the original image and the Gaussian filter. The filter is a square and its size is calculated by 2[2*σ* + 1]. It is possible to remove high-frequency components of the speckle noise by the convolution operation with a fringe image. The frequency characteristic of filter is determined by standard deviation σ. Figure 3 shows frequency characteristics for each sigma value. Mean interval of fringe appeared in Figure 2a is roughly 70 pixels. The smallest fringe interval obtained in this study is 20 pixels as discussed in the later sections. In other words, the number of fringes obtained in this study is around 17 per image length. According to Figure 3, the standard deviation at σ=4 is low enough to cover the measured fringe resulting from the deformation. Figure 2b shows the filtered image at the σ=4. The resultant image shows that Gaussian filter can remove the high frequency noises without losing the main fringe information.

- Step (ii): Phase determination with partial differential image

To determine the phase at positions of “dark fringe”, we devised a new algorithm using partial differentiation as follows. The filtered speckle intensity can be calculated as follows:

(9) $$I_{filtered}=4A^{2}\left|\sin\frac{\Delta \phi }{2}\right|$$

Then, the partial differentiation in the direction *y* of Equation (9) can be written as follows:

(10) $$\frac{\partial I_{filtered}}{\partial y}=\{\begin{array}{cc} 4A^{2}\frac{\Delta \phi^{\prime }}{2}\cos\frac{\Delta \phi }{2} & 2n\pi <\frac{\Delta \phi }{2}<(2n+1)\pi \\ -4A^{2}\frac{\Delta \phi^{\prime }}{2}\cos\frac{\Delta \phi }{2} & (2n-1)<\frac{\Delta \phi }{2}<2n\pi \end{array}$$

**Figure 4:**
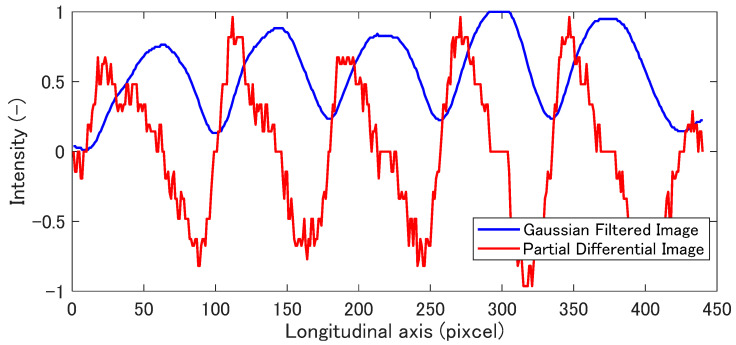
Intensity distribution in the direction *x*.

It should be noted that ∂I/∂y switches discontinuously positive to negative at the phase difference, Δϕ=2nπ. Figure 4 shows the intensity profile of Figure 2b in the direction *y* and its partial derivative obtained by Equation (9). The intensity is centerline of the direction *x*. Since the intensity change in the derivative curve becomes discontinuous, the phase can be easily discriminated. Figure 2c shows the image obtained by applying the Equation (10) to Figure 2b. The result is scaled in 256 gradation.

- Step (iii): Binarization

The partially differentiated intensity is normalized in integer value from −1 to 1 as shown in Figure 4. In the differentiated profile, the position where the value is zero means the valley of original intensity profile, “the dark fringe”. The differentiated image is binarized with a threshold of 0, in other words, all color values under 0 become 0, while all others become 1. This process detects the dark fringe as a boundary of the region without depending on the bias of intensity of the fringe pattern. The binarized image is obtained as shown in Figure 2d.

- Step (iv): Morphological processing

Figure 2d still has tears and minute regions. In order to extract only the main fringe region, the binarized image was subjected to morphological process of erosion and dilation. Figure 2e shows the morphological processed image. By detecting the boundary of the black and white area of the processed image, the valley part of the fringe pattern can be extracted. The colored line in Figure 2e is a plot of the border where the area changes from black to white for each area.

- Step (v): Detection fringes and interpolation

The border line of the area from Figure 2e is looks discontinuity, however, the actual deformation of the specimen should occur continuously. In the previous works [1,2,3,4], we demonstrated that the elastic/plastic deformation can be expressed based on the field theory. The deformation process will be discussed based on the second order derivative function of the displacement. Thus, it is accurate enough to fit the curve with a quadratic function. Figure 2f shows fitted lines overlaid in the original speckle image. Regarding the displacement in the tensile direction, the phase at the fixed end of specimen was set to 0, and the order of fringe, n was indexed forward to the crosshead side. Then, the phase values between the dark fringes were interpolated by piecewise cubic hermite interpolating polynomial (PCHIP) algorithm [12]. Regarding the displacement in the widthwise direction u, the fringe order n was indexed from the left end to the right end, followed by the interpolation in the same manner of tensile direction. Assuming that the displacement in the direction x is bilaterally symmetric, all phase values were shifted so that the phase at the center coordinates of the specimen is zero. Consequently, two-dimensional displacement vector ξ(u,v) is obtained. Strain and rotation tensors are defined as follows:

(11) $$E=\left[\begin{array}{cc} \varepsilon_{xx} & \varepsilon_{xy}\\ \varepsilon_{yx} & \varepsilon_{yy} \end{array}\right]=\left[\begin{array}{cc} \frac{\partial u}{\partial x} & \frac{1}{2}\left(\frac{\partial u}{\partial y}+\frac{\partial v}{\partial x}\right)\\ \frac{1}{2}\left(\frac{\partial v}{\partial x}+\frac{\partial u}{\partial y}\right) & \frac{\partial v}{\partial y} \end{array}\right]$$

(12) $$\Omega =\frac{1}{2}(\Delta \times \xi )=\left[\begin{array}{cc} 0 & \frac{1}{2}\left(\frac{\partial u}{\partial y}-\frac{\partial v}{\partial x}\right)\\ \frac{1}{2}\left(\frac{\partial v}{\partial x}-\frac{\partial u}{\partial y}\right) & 0 \end{array}\right]$$

Here, assuming a two-dimensional plane strain condition, the displacement along z axis, w=0, ∂u/∂z=∂v/∂z=0. The rotation vector ω in the plane strain condition is given as follows.

(13) $$\omega =\left[\begin{array}{c} \omega_{x}\\ \omega_{y}\\ \omega_{z} \end{array}\right]=\left[\begin{array}{c} 0\\ 0\\ \frac{1}{2}\left(\frac{\partial v}{\partial x}-\frac{\partial u}{\partial y}\right) \end{array}\right]$$

## 3. Deformation Process Observed in the Tensile Test

### 3.1. Material and Specimen

**Figure 5:**
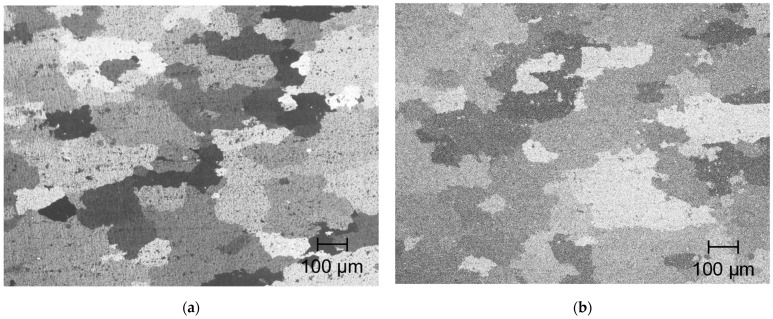
Optical micrographs of (**a**) 7075-T6 specimen and (**b**) 7075-annealed specimen.

**Figure 6:**
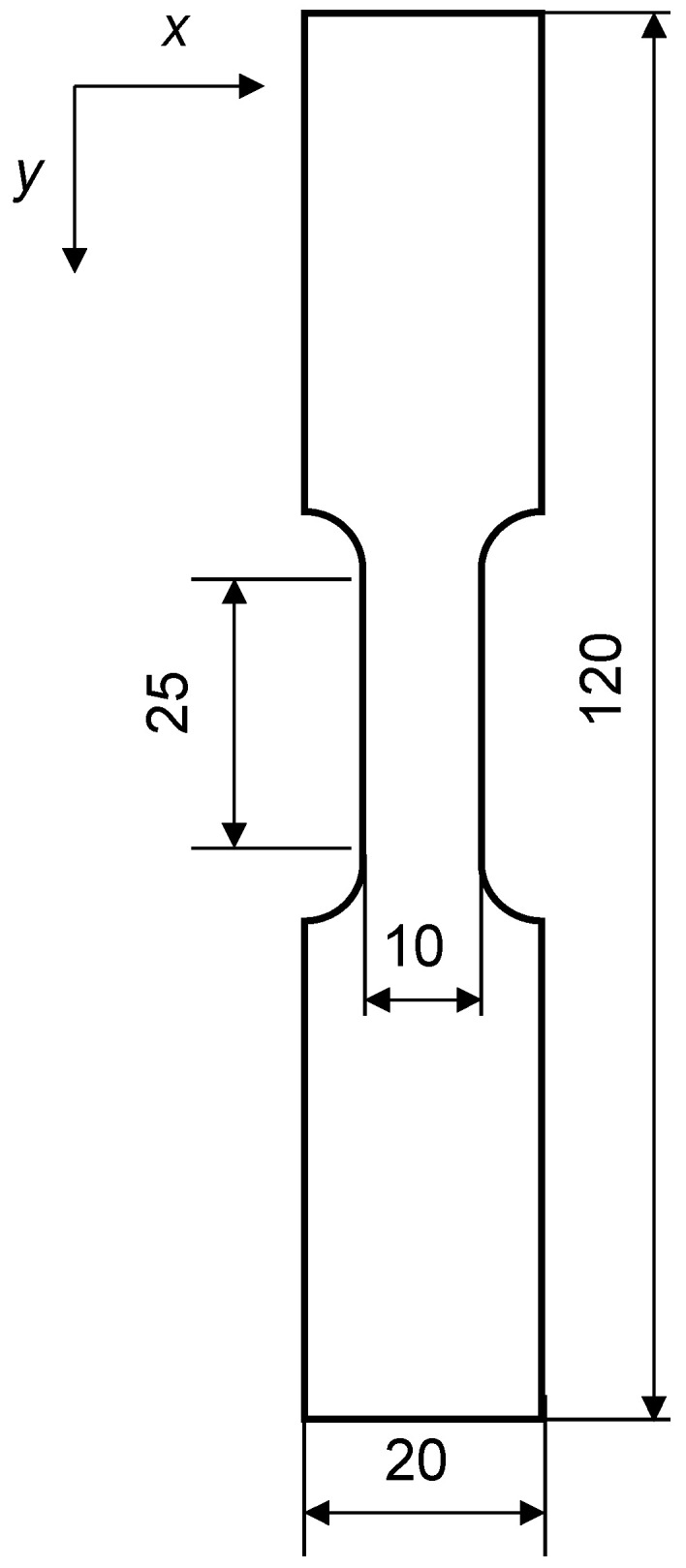
Shape of the Specimen.

The material used in this study was a sheet of industrial Al–Zn–Mg–Cu alloy (AA7075) with 5 mm thick. The chemical composition of alloy is shown in Table 1. To investigate the effect of matrix hardness on the deformation process, the following two types of heat treatments were employed. First, the material was solution treated at 480 °C for 2 h and hardened at 120 °C for 24 h up to peak hardness (7075-T6 alloy). In the microstructural view, the solute atoms form precipitates, resulting in high yield strength. The T6 treated alloy was subsequently over aged at 400 °C for 30 min. The matrix is softened by coarsening the precipitates (7075-annealed alloy). The optical micrographs of specimens are shown in Figure 5. Mean grain sizes of two specimens are roughly 150 µm. As shown in Figure 6, the materials were cut into tensile specimens of 10 mm in gauge width and 25 mm in gauge length.

### 3.2. Experimental Results

**Figure 7:**
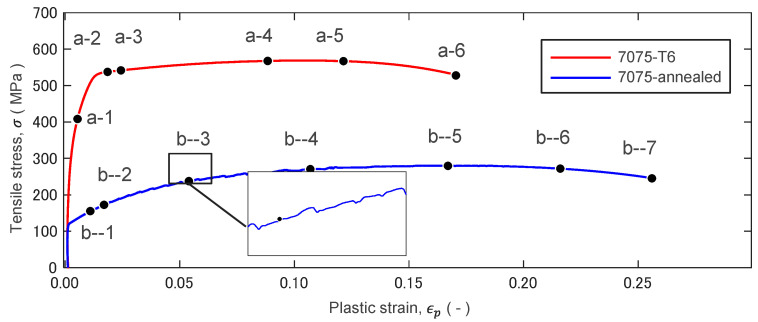
Stress–strain curves of 7075-T6 and 7075-annealed alloys.

**Figure 8:**
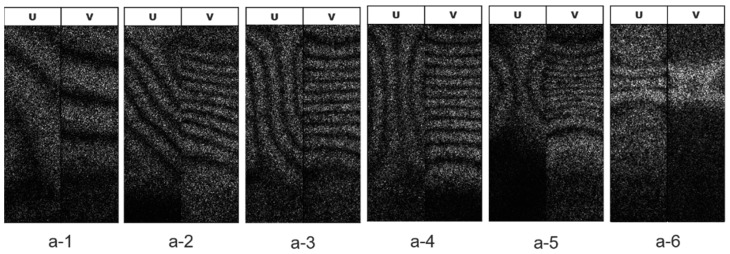
Evolution of fringe patterns observed in tensile test of 7075-T6 alloy. Images (a-1 to a-6) show the patterns observed at the strains marked in Figure 7.

**Figure 9:**
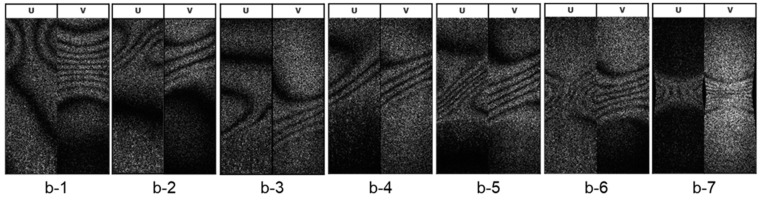
Evolution of fringe patterns observed in tensile test of 7075-annealed alloy. Images (b-1 to b-7) show the patterns observed at the strains marked in Figure 7.

Figure 7, Figure 8 and Figure 9 show stress–strain curves (S-S curve) of 7075-T6 alloy and 7075- annealed alloy, and the fringe patterns observed at several strain levels. The fringe images shown in the figure represent respectively.

#### 3.2.1. 7075-T6 Alloy

7075-T6 alloy shows a homogenous plastic deformation. Figure 8a-1–a-6 respectively show the fringe patterns observed at the strains marked in Figure 7. At the stress under 500 MPa, the fringe pattern of longitudinal displacement *v* (*v*-fringe) is almost vertical to the tensile axis and an equal interval in all area of specimen. The *v*-fringes start to curve and concentrate to a neck of specimen (Figure 8a-1). Some number of vertical fringes to the tensile axis appears in the displacement *u* (*u*-fringes), indicating that the yielding initiated with shear and rigid body rotation. At the yielding the number of fringes increases, while the fringes with equal interval shows homogenous deformation. The deformation shows macroscopically homogeneous (Figure 8a-2–a-4). At the maximum load of 550 MPa in the S-S curve, the *v*-fringes concentrated to center of specimen with curving of *u*-fringes and necking of specimen subsequently begin (Figure 8a-5). The fringe concentration forms a bright pattern, resulting in a fracture at the center of bright pattern.

#### 3.2.2. 7075-Annealed Alloy

7075-annealed alloy shows inhomogeneous plastic deformation characterized by a serrated curve as shown in Figure 7 in blue. The deformation process is completely different from that observed in the 7075-T6 alloy. The serration phenomenon is closely related to instability of the plastic deformation, and it is well known as the Portevin-Le Chatelier (PLC) effect. The fringes in the elastic range show similar patterns to 7075-T6 alloy. The fringe starts to concentrate just after the yielding (Figure 9b-2). The concentrated fringes exhibit a drift motion along the parallel part of specimen. The v-fringes and u-fringe subsequently tilt to an angle of 45°, forming a shear band as shown in Figure 9b-2–b-3. The shear band drifts along the parallel part of specimen and disappears at the neck part Figure 9b-4–b-6. The serrated curve is clearly seen in the S-S curve in the plastic strain $\varepsilon_{p}>0.05$. The plastic deformation progresses with the drift motion of the shear band. The activity of shear band repeats until near the maximum stress. At the maximum stress in the 7075-annealed alloy (Figure 9b-7), the shear band activity becomes stationary at the center of specimen, then the material fails.

## 4. Discussion

In this section, we discuss the deformation behaviors observed in the present experiment based on the field theoretical interpretation of deformation and fracture. The field theoretical behavior of the deformation in the transition from the pre-yield stage to the post-yield stage, the plastic deformation dynamics in association with the mobile dislocation dynamics, and the transition from the late plastic stage to the fracturing stage in association with the corresponding wave dynamics are addressed. Through the present study we have found an interesting contrast between T6 and annealed in their deformation behaviors. The former exhibit more brittle-material like behavior and the latter more ductile-material like behavior in the transition to the fracturing stage. We will discuss the contrast from the field theoretical viewpoint.

### 4.1. General Arguments of Deformation and Fracture Based on Field Theory

#### 4.1.1. Equation of Motion

According to the field theory, the material force acting on a unit volume of solid under deformation is given as follows:

(14) $$\rho \frac{\partial v}{\partial t}=(\lambda +2G)\nabla^{2}\xi -G(\nabla \times \omega )-\sigma_{0}\rho (\nabla \cdot v)v-(E\delta x\Delta x)(\partial_{x}^{4}\xi )$$

Here ρ is the density of the deforming material, v is the particle velocity vector, ξ is the particle displacement vector, G is the shear modulus, ω is the rotation vector, $\sigma_{0}$ is the material constant representing the degree of viscosity, E is the Young’s modulus, Δx is the width of a concentrated strain and δx is the small width that borders the concentrated strain from the rest of the specimen. (See below under “Solitary wave” for more descriptions about Δx and δx). On the right-hand side of this equation, the first term represents the continuum mechanical longitudinal elastic force, the second term the shear elastic force associated with volumetric rotation, the third term the plastic energy dissipative longitudinal (velocity damping) force, and the last term the elastic force associated with nonuniform normal strain distribution.

#### 4.1.2. Rotational Nature of Plasticity

It is generally true that in the post-yield regime the material still possesses elasticity. This is evidenced by the fact that a material about to fracture due to a tensile load shrinks back to some extent if the load is removed. The field theory characterizes the yield phenomenon as the shear instability. When an elastic material yields, the elastic-deformation dynamics shifts from the one dominated by the longitudinal elasticity to the one dominated by the shear elasticity. In terms of Equation (14), this transition is represented by the shift that the second term dominates over the first term on the right-hand side, i.e., (∇×ω) becomes visible in the differential displacement field.

#### 4.1.3. Irreversibility of Plasticity

The irreversibility of plasticity is represented by the longitudinal energy dissipative mechanism represented by the third term on the right-hand side of Equation (14). This energy dissipative mechanism and the fracture mechanism as its final stage can be argued with the analogy to the electrical breakdown of gas media. Formulaically, the term (∇⋅v) resembles the electric charge density $\rho_{e}=\varepsilon (\nabla \cdot E)$ if we replace the velocity vector v with the electric field vector E. Here ε is the electric permittivity. The electric conduction current density is given as $j=\rho_{e}W_{d}=\varepsilon (\nabla \cdot E)W_{d}$ where $W_{d}$ is the drift velocity proportional to the electric field via the electric mobility $\mu_{e}$ as $W_{d}=\mu_{e}E$. Thus, $j=\varepsilon (\nabla \cdot E)\mu_{e}E$. According to Maxwell electrodynamics, the conduction current density is proportional to the electric field via the material constant known as the conductivity as j=σE. Equating these two expressions, we find $j=\sigma E=\varepsilon (\nabla \cdot E)\mu_{e}E$, hence,$\sigma =\mu_{e}\varepsilon (\nabla \cdot E)$. We can view the term $\sigma_{0}\rho (\nabla \cdot v)$ as corresponding to $\mu_{e}\varepsilon (\nabla \cdot E)$, and the entire term $\sigma_{0}\rho (\nabla \cdot v)v$ as resembling the conduction current density $j=\varepsilon (\nabla \cdot E)\mu_{e}E$. When conduction current flows under the influence of the electric field, the energy is dissipated as heat. This energy loss is known as Ohmic loss. We can interpret the plastic energy loss mechanism as resembling the Ohmic loss mechanism. Based on this analogy, we call the quantity ∇⋅v as the deformation charge density.

### 4.2. Experimental Observations

#### Increase in ∇×ω and Localization of ∇⋅v with Development of Deformation.

**Figure 10:**
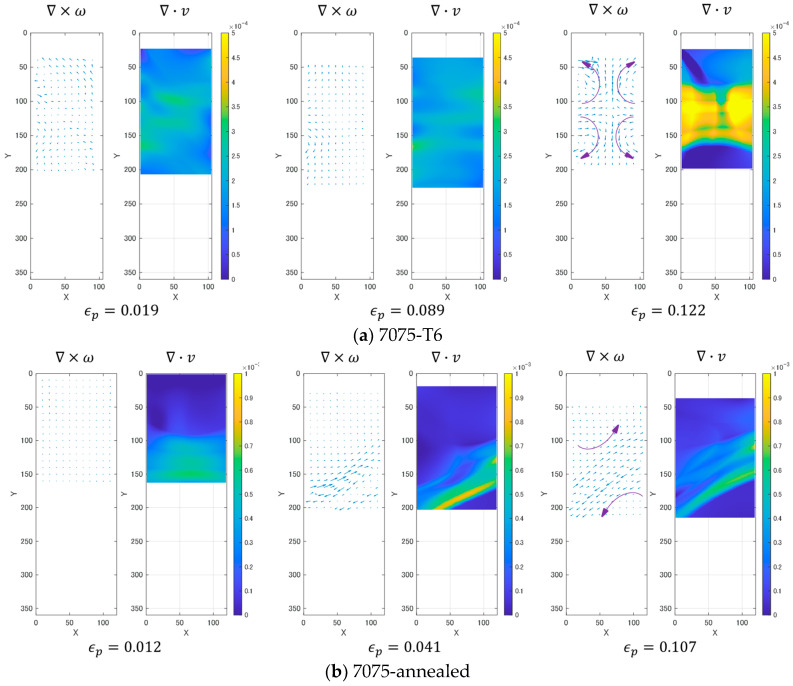
Typical ∇×ω map in the plastic deformation processes.

Figure 10 shows the changes in the ∇×ω field and the deformation charge density ∇⋅v observed with the development of plastic deformation. Here, $\varepsilon_{p}$ denotes the plastic strain, and for each plastic strain the quiver plot displays the two-dimensional ∇×ω vector and the surface plot displays the charge density ∇⋅v. These fields are evaluated on the -plane based on the fringe analysis described in Section 2.2. With the increase in the plastic strain, the ∇×ω field becomes less uniform. At the same time, the charge distribution is localized in a certain region of the specimen where the charge density increases.

While commonly showing the above-mentioned change in the ∇×ω field and the charge density ∇⋅v, the T6 and annealed specimens exhibit certain differences. The T6 specimen exhibits a more horizontally symmetric feature, both in the ∇×ω field, and in the charge distribution as compared with the annealed specimen. Figure 10a shows that when the charge density is concentrated near the vertical center, the T6 specimen still shows horizontally symmetric distribution in both the ∇×ω field and the charge density ∇⋅v. The concentrated charge distribution shows an X-like shape. Contrastively, the annealed specimen exhibits a horizontally asymmetric feature in the ∇×ω and ∇⋅v patterns, as the ∇×ω field starts to be less uniform. The concentrated charge distribution runs across the specimen width forming a slanted band structure.

**Figure 11:**
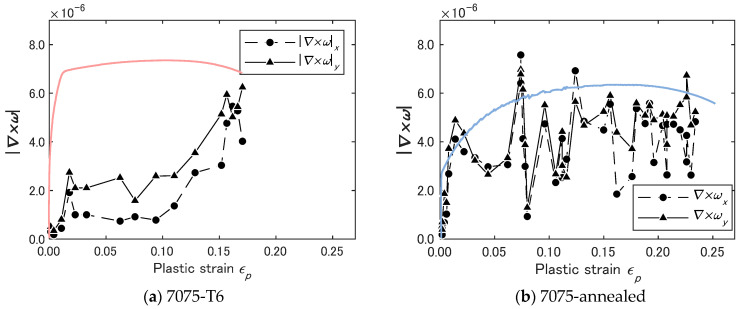
Variation of |∇×ω| (average over shear band zone) during the tensile test.

The ∇×ω field shows an increase with the development of plastic deformation. Figure 11 plots the magnitude of ∇×ω vector averaged over the approximately 8 × 8 mm area where the ∇⋅v pattern is drawn in Figure 10. The average |∇×ω| is plotted as a function of plastic strain $\varepsilon_{p}$. From the yield point (corresponding to $\varepsilon_{p}=0$ in Figure 11) onward, the average |∇×ω| increases sharply. After the initial sharp rise, in both specimens commonly, the average |∇×ω| decreases at a slower rate to a minimum and resumes increasing to the highest peak at approximately the same rate as it decreases from the first peak.

The two specimens show difference in the temporal behavior of the average as well. In the case of the T6 specimen, the average |∇×ω| shows the above-mentioned minimum only once as seen in Figure 11a. On the other hand, the annealed specimen shows multiple minima, exhibiting the oscillatory behavior observed in Figure 11b.

Careful analysis on the ∇×ω field and the ∇⋅v distribution in Figure 10 reveals that the concentrated charge density ∇⋅v appears next to the region where the ∇×ω field grows. In the case of the T6 specimen, a symmetric pattern of the concentrated charge density appears between a pair of grown ∇×ω. The quiver plot for $\varepsilon_{p}=0.122$ in Figure 10a shows an example of such a pattern. The arrows inserted in the quiver plot illustrate the approximate direction of the ∇×ω vectors, which represents the pattern of shear force in the area. We can interpret that the X-shaped pattern of ∇⋅v is formed at the boundary of the vertical pair of the curly ∇×ω field. On the right half of the specimen, the material on the upper side of the boundary rotates clockwise and the lower side counterclockwise. On the left half of the specimen, the pattern is opposite to the right half. The upper side of the boundary rotates counterclockwise and the lower side clockwise. This combination of four rotations forms a horizontally inward velocity. It is possible to interpret this as the indication that the specimen starts to undergo necking. In the stress–strain curve shown in Figure 7, $\varepsilon_{p}=0.122$ is marked a-5. From this point onward, the stress decreases monotonically. In this process, the symmetric ∇⋅v pattern keeps increasing the degree of localization, turning an X-shaped pattern (a-6). Macroscopically, this indicates an increase in the degree of necking.

The annealed specimen displays a different behavior. The slant band structured pattern of ∇⋅v appears along the boundary of the same rotations of ∇×ω field. Figure 10b $\varepsilon_{p}=0.107$ indicates that on the upper and lower sides of the boundary, the rotations are counterclockwise. It is interesting to compare this pattern with the four rotations observed in the T6 specimen in Figure 10a $\varepsilon_{p}=0.122$. We can interpret that the two rotations observed in the annealed specimen correspond to the upper left and lower right rotations observed in the annealed specimen. In an earlier stage, the annealed specimen exhibits the same symmetric type fringe pattern as the T6 specimen (see Figure 9b-1). It is likely that when the plastic strain develops to the level where the slant band structure appears, the clockwise rotations observed in the early stage on the upper right and lower left of the boundary disappear. As a result, the pair of the counterclockwise rotations exert strong shear force along the boundary.

We can interpret the above-mentioned transition from the “X”-shaped to the slant band structure as being associated with the transition from the normal force dominating regime to the shear force dominating regime. In the early stage of deformation when the deformation is in the linear elastic regime, the longitudinal elastic force represented by the first term on the right-hand side of Equation (14) is dominant. With the development of plastic deformation, the longitudinal resistant force becomes less effective, and instead, the shear resistant force represented by the second term on the right-hand side of Equation (14) becomes more active. It is naturally understood that at a certain point, the shear force dominates over the longitudinal force. This transition of dominant resistant force mechanism accompanies two events. The first is a stress drop. This is because the shear modulus is lower than the longitudinal (Young’s) modulus. The second is the formation of shear force across the boundary over the entire width of the specimen.

### 4.3. Activity of Shear Band

#### 4.3.1. Shear Elastic Dynamics in Shear Band

Figure 9, Figure 10 and Figure 11 indicate that the increase in ∇×ω is accompanied by a concentration of ∇⋅v. When ∇×ω grows to a certain level, the charge ∇⋅v takes the form of band structure. In the case of T6 the band is in an “X”-shape and its appearance coincides with the necking. In the case of annealed it is more a shear band like (running from one side to the other side of the specimen as a slant line) until the final fracture. The slant band structures run at the boundary of two developed ∇×ω fields.

The fringe pattern observed with the ESPI setup represents the spatial dependence of the differential displacement occurring during the time step Δt used for the image subtraction. Thus, each fringe can be interpreted as the contour of the velocity measured in the unit of Δt, and thus proportional to the velocity in the unit of m/s, dξ/dt. When the fringes are linear parallel, we can express the fringe pattern as follows:

(15) $$\frac{d\xi }{dt}(u,v)=\left[\begin{array}{c} c_{11}x+c_{12}y\\ c_{21}x+c_{22}y \end{array}\right]$$

Here the linearity allows us to express u and v as a linear function of x and y where $c_{11}\ldots c_{22}$ are all constant, and the constant of proportionality between the velocity in the unit of Δt and m/s is absorbed in $c_{11}\ldots c_{22}$. The fact that the linear fringes are parallel allows us to use the same constants $c_{11}\ldots c_{22}$ for the entire boundary region.

(16) $$\omega_{z}=\frac{\partial v}{\partial x}-\frac{\partial u}{\partial y}=\int (c_{21}-c_{12})dt$$

Equation (16) indicates that $\omega_{z}$ is independent of the space coordinates, which in turn indicate that ∇×ω = 0 as follows:

(17) $$\nabla \times \omega =\left(\frac{\partial \omega_{z}}{\partial y}-\frac{\partial \omega_{y}}{\partial z}\right)\hat{x}+\left(\frac{\partial \omega_{x}}{\partial z}-\frac{\partial \omega_{z}}{\partial x}\right)\hat{y}+\left(\frac{\partial \omega_{y}}{\partial x}-\frac{\partial \omega_{x}}{\partial y}\right)\hat{y}=\frac{\partial \omega_{z}}{\partial y}\hat{x}-\frac{\partial \omega_{y}}{\partial x}\hat{y}$$

The condition ∇×ω = 0 indicates that one of the pre-fracture criteria holds in the shear band region. Notice that the quiver plots in Figure 10 indicate that the regions on both sides of the shear band undergo differential rotations exerting the horizontal resistive force represented by $\partial \omega_{z}/\partial y\hat{x}$ and the vertical resistive force represented by $\partial \omega_{z}/\partial x\hat{y}$. The formation of a shear band can be interpreted as that these differential rotational forces cause strong, localized shear force along the boundary and that consequently the shear banded area exhibits the pre-fracture condition.

While developed shear forces are present above and below a shear band, the above observation that ∇×ω = 0 in the shear band region indicates that this region does not exert the resistive force. This is the basis of the claim that a pre-fracture criterion is met in a shear band. Figure 11b indicates that this type of pre-fracture condition is recoverable. The field theory interprets this recovery as a phenomenon analogous to the spark discharge. Although the recovery mechanism from this pre-fracture condition has not been clarified, it is possible that some sort of atomic rearrangement in association with motions of mobile dislocations underlies the recovery (see a section below).

#### 4.3.2. Shear Band as a Constant Charge ∇⋅v

The approximately parallel and linear fringe pattern inside a shear band indicates that the differential displacement inside the band represents normal strain in a direction perpendicular to the fringes. From Equation (15), we can express the shear band fringe pattern as follows, and thereby interpret it as a constant deformation charge ∇⋅v.

(18) $$\nabla \cdot v=\frac{\partial }{\partial t}\left(\frac{\partial u}{\partial x}+\frac{\partial v}{\partial y}\right)=c_{11}+c_{22}=const(damping\;coefficient)$$

**Figure 12:**
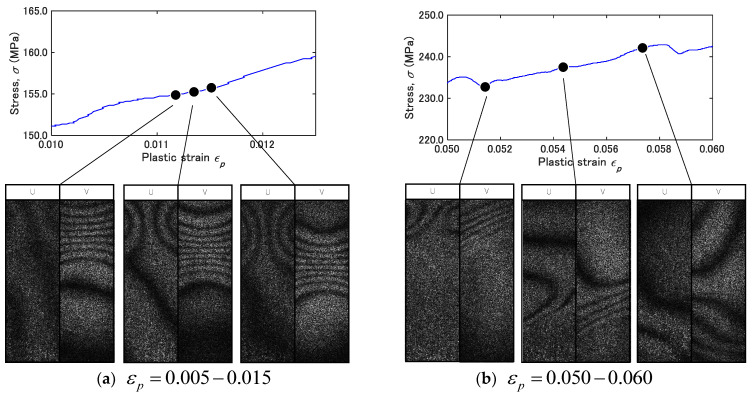
Shear band activity in 7075-annealed alloy observed in the strain range of (**a**) $\varepsilon_{p}=0.005-0.015$ and (**b**) $\varepsilon_{p}=0.050-0.060$.

Under the condition that $c_{11}$ and $c_{22}$ are constant, the shear band drifts keeping its shape. This is a constant movement of a deformation charge. Figure 12 shows fringe patterns in the annealed specimen that we can interpret as representing deformation charges. The patterns shown in Figure 12a are observed in a stage earlier than (b). The plots above these fringe images are the stress–strain characteristics for the respective stages. The locations on the stress–strain curve at which each image is observed are indicated by markers and the lines connecting with the fringe patterns. Careful observation of the strain-stress curve reveals the following aspects: (1) The three images in Figure 12a are not associated with a sharp stress drop; (2) The two left images in (b) are observed after a sharp stress drop and the rightmost images is observed after the next stress drop. Of these six images, the ones observed after a stress drop exhibit a shear band like structure. The one after the stress rise does not show a band structure. These aspects indicate that the formation and motion of the shear-band like fringe pattern (or the deformation charge) are associated with a stress drop.

This observation is consistent with the above-argued stress drop resulting from the transition of longitudinal-force dominant to shear-force dominant resistive mechanism. Macroscopically, this sequence of stress drop and resumption can be interpreted as representing the serration.

#### 4.3.3. Connection to Mobile Dislocations and Serrations

Figure 10b indicates that the shear force ∇×ω vectors on the opposite sides of a shear band curls oppositely as indicated by curved arrows inserted in the rightmost quiver plot for each specimen. The mutually opposite curly vector fields exert strong differential shear force along the boundary. This differential shear force can be interpreted as the frictional force that drives mobile dislocations.

Based on the above interpretation that the differential shear force drives mobile dislocations, we can make the following arguments. Mobile dislocations are stopped by obstacles such as the grain boundary. This interpretation is consistent with the experimental observation that the speed of shear band exhibits the same time dependence as that of mobile dislocations [4]. Being driven by a frictional force, the mobile dislocations cause energy dissipation as they move. This is consistent with the field theoretical interpretation that the resistive force represented by $\sigma_{0}\rho (\nabla \cdot v)v$ term in Equation (14) is velocity damping force. The energy dissipative dynamics of mobile dislocations accounts for the plastic irreversibility. Once mobile dislocations reach a side of the specimen, the left side in the case of $\varepsilon_{p}=0.107$ in Figure 10b, the dislocations do not move any longer. This explains the end of shear band drift and resumption of stress rise as the specimen recovers from the partial pre-fracture. The rightmost image and corresponding stress–strain characteristics exhibit that the disappearance of shear band like fringe pattern and appearance of curved fringe patterns accompanied by a fringe pattern.

#### 4.3.4. Solitary Wave

The field theory explains that the movement of a shear band corresponds to a motion of a solitary wave [4]. The condition ∇×ω=0 makes to rewrite Equation (14) in the following form. Note that at this stage the linear elastic force term $(\lambda +2G)\nabla^{2}\xi$ is inactive as well.

(19) $$\rho (\partial_{t}v)=\sigma_{0}\rho v(\partial_{x}v)-\frac{\left(E\delta x\Delta x\right)}{c_{s}}(\partial_{x}^{3}v)$$

Here, the following replacement is used to rewrite Equation (14) into Equation (17)

(20) $$\partial_{x}v=\frac{\partial \xi }{\partial x}=\frac{\partial \xi }{\partial t}\frac{dt}{dx}=\frac{1}{c_{s}}\frac{\partial \xi }{\partial t}$$

Equation (17) is known as Korteweg-de Vries (KdV) equation and has a solution in the following form:

(21) $$v=a{\mathrm{sech}}^{2}\left(b\left(x-c_{w}t\right)\right)$$

(22) $$c_{w}=\frac{\sigma_{0}a}{3}$$

(23) $$b=c_{w}\sqrt{\frac{\rho }{4E\delta x_{s}\Delta x_{s}}}$$

Here, a is the amplitude of the solitary wave, b is a constant decided by the material’s property and the shape of the shear band, and $c_{w}$ is the propagation velocity of the solitary wave.

**Figure 13:**
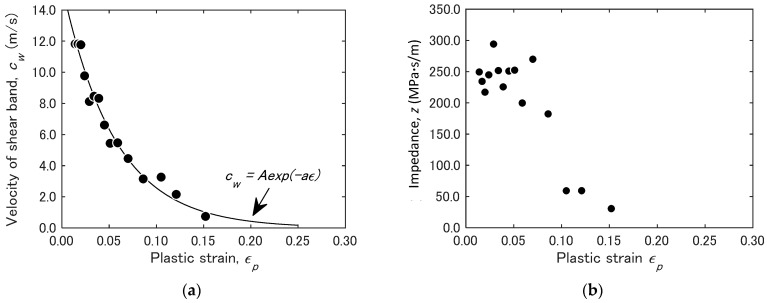
Velocity and impedance of shear band in 7075-annealed alloy.

It is interesting to discuss the behavior of shear band observed in the experiment in conjunction with the above expressions of the solitary wave. Figure 13a shows the change of velocity of shear band observed during the tensile test. The band velocity shows an exponential decrease similar to the decay in the amplitude of the |∇×ω| observed in Figure 11b. Equation (22) indicates that the solitary wave velocity is proportional to the amplitude of the solitary wave. As the deformation develops toward the final fracture, the level of energy dissipation increases, damping the particle movement. This makes the amplitude of the velocity solitary wave decrease. The exponential decrease in $c_{w}$ is understandable because velocity damping causes exponential decrease in the displacement and velocity fields. The same damping mechanism explains the exponential decay of the |∇×ω| amplitude in Figure 11b.

Now let us discuss the behavior of the solitary wave in terms of the motion of the deformation charge ∇⋅v. From the analogy to the electromagnetic field, we can argue that the motion of the deformation charge corresponds to the conduction current. For a given electric potential, conduction current flows depending on the resistance (impedance). In the electric field, when the electric charge density is concentrated in a local region, the increase in the charge density lowers the impedance, which further increases the local charge density. Once this positive feedback mechanism dominates, the gas medium electrically breaks down quickly. It is expected that in the deformation field the impedance decreases as well by a similar positive feedback mechanism [4]. Since we are dealing with displacement wave, it is reasonable to discuss the acoustic impedance given in the following form.

(24) $$z=\frac{\kappa }{v_{p}}=\frac{d\sigma /d\varepsilon }{c_{w}}$$

Here, κ is the stiffness of the medium and $v_{p}$ is the wave velocity. In the present context, the stiffness can be evaluated as dσ/dε and the wave velocity is $c_{w}$.

Figure 13b plots the impedance evaluated by substituting dσ/dε into κ and $c_{w}$ into $v_{p}$ in Equation (24). As expected, impedance decreases monotonically with the plastic strain $\varepsilon_{p}$. Figure 13a indicates that $c_{w}$ also decreases with $\varepsilon_{p}$, which tends to increase impedance z in Equation (24). The fact that the impedance decreases despite the decrease in $c_{w}$ on the denominator of Equation (24) indicates that the decrease in the stiffness is greater than the increase in $c_{w}$. As discussed above, the decreases in the wave velocity $c_{w}$ can be attributed to the increase in the damping characteristics of the material. The similar damping has been observed in acoustic measurement [13]. The velocity acoustic wave in solid metals is dependent on the dislocation structure. The progress of plastic deformation with the increase of dislocation density results in the damping of acoustic velocity. The observed monotonic decrease in impedance indicates that the stiffness decreases faster than the increase in the damping effect.

#### 4.3.5. Comparison of T6 and Annealed Specimens

It is interesting to compare the T6 and annealed specimens for various behaviors. Figure 6 clearly shows that the T6 specimen exhibits more brittle behavior in the stress–strain characteristics and the annealed specimen exhibits more ductile behavior. This contrast can be observed in other features of the two specimens.

First, look at the difference in ${\left(\nabla \times \omega \right)}_{x}$ and ${\left(\nabla \times \omega \right)}_{y}$. These are the x- and y-component of shear displacement. Figure 11a indicates that in the T6 specimen is factor of two to three greater than ${\left(\nabla \times \omega \right)}_{x}$. When ∇×ω reaches the maximum value, this relation of ${\left(\nabla \times \omega \right)}_{x}$ to ${\left(\nabla \times \omega \right)}_{y}$ becomes unclear. The ratio of these two quantities essentially represents Poisson’s effect. It makes sense that as ∇×ω reaches the maximum value, the deformation is characterized more by plasticity than elasticity, hence the Poisson’s effect becomes less prominent. The argument that the annealed exhibits more ductile behavior is consistent with the fact that the annealed specimen shows more serrations in the stress–strain characteristics in Figure 7. Figure 11b indicates that the annealed specimen shows that ${\left(\nabla \times \omega \right)}_{y}$ is somewhat greater than ${\left(\nabla \times \omega \right)}_{x}$ but the difference is not as clear as the T6 case. Being more ductile material, the annealed specimen does not show Poisson’s effect at the same level as the T6 specimen. Figure 11 also shows that the T6 specimen exhibits the increase in ∇×ω once. On the other hand, the annealed specimen shows the increase and decrease in ∇×ω several times repetitively. The discussions made in the above sections indicate that each cycle of increase in ∇×ω corresponds to the motion of mobile dislocations to a side of the specimen. The observed contrast in the pattern of ∇×ω increase/decrease characterizes that the annealed specimen is more ductile.

Another interesting observation is that the concentrated charge density pattern of the T-7 specimen regains horizontal symmetry toward the stage of final fracture as presented by Figure 9b-7 fringe image. It is likely that the symmetric feature in the charge density is the natural behavior of the specimen as the applied load and the specimen geometry is symmetric. It is the dislocation’s motion that causes the observed asymmetric behaviors of the deformation field. Towards the end of the ductile deformation, the dislocations stop their activity, hence the deformation field regains the symmetric feature. Being a more brittle material, the T6 specimen does not exhibit this dislocation-induced asymmetric behavior in the deformation field.

## 5. Conclusions

The plastic deformation behavior of 7075-T6 and 7075-annealed has been visualized using ESPI and discussed based on the field theory. The following conclusions are obtained:

1. The proposed method for the fringe analysis using image processing can detect the fringe contours without a phase-stepping method. The dynamic deformation behavior of 7075-alloys can be evaluated using this method.
2. With the increasing of the plastic strain, the ∇×ω field becomes less uniform. At the same time, the charge distribution is localized in a certain region of the specimen where the charge density increases. These transitions from symmetric to asymmetric deformation field are caused by transitions from longitudinal-force dominant resistive mechanisms to shear-force dominant mechanisms.
3. The deformation localization is closely related to the shear elastic dynamics. In microscopic view, the plastic deformation is initiated via driving the mobile dislocation. The shear force (∇×ω) vectors on the opposite sides of a shear band curls oppositely. The mutually opposite curly vector fields exert strong differential shear force along the boundary. This differential shear force can be interpreted as the frictional force that drives mobile dislocations.
4. The T6 specimen exhibits more brittle behavior in the stress–strain characteristics and the annealed specimen exhibits more ductile behavior. The observed contrast in the pattern of ∇×ω increase/decrease characterizes that the T6 specimen is more brittle and annealed is more ductile.
5. The fringe patterns of the T6 and annealed specimens show similar characteristics in the initial stage, differ in the progressive stage of deformation, and show similarity again in the final stage. This is because the dislocations are not active in the initial and final stages.

## Figures and Tables

**Table 1:** Chemical composition of AA7075 (mass %).

Alloy	Si	Fe	Cu	Mn	Mg	Cr	Zn	Ti
7075	~0.40	~0.50	1.2~2.0	~0.30	2.1~2.9	0.18~0.28	5.1~6.1	~0.20

**Figure A1:**
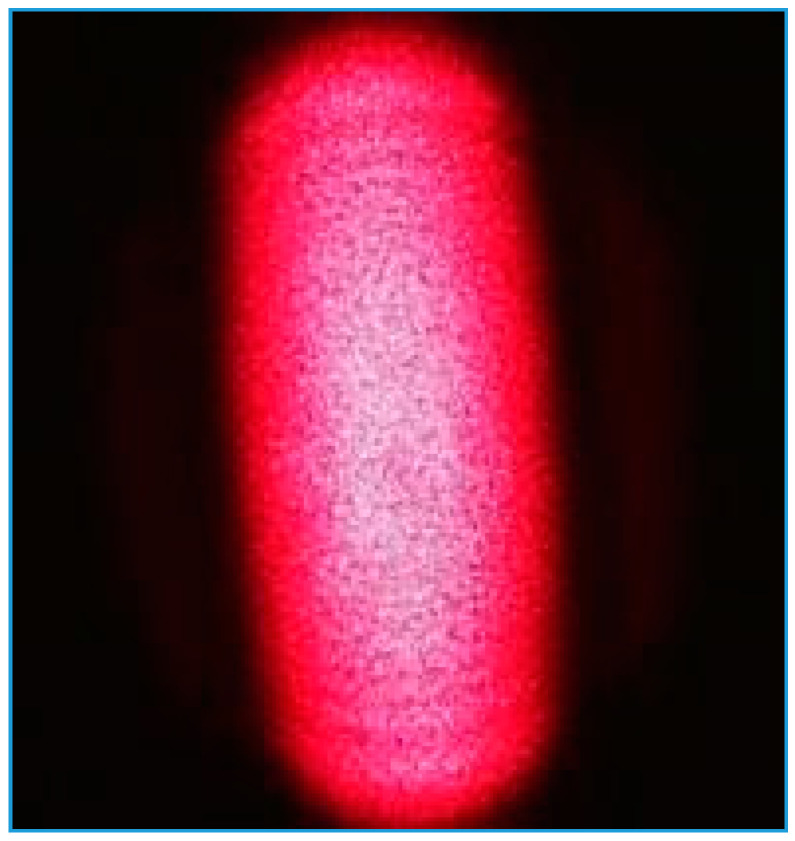
Laser speckle pattern.

**Figure A2:**
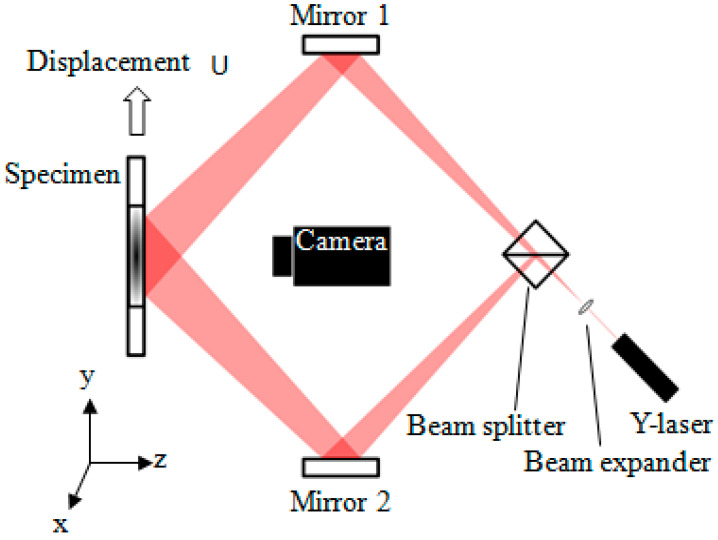
Optical system of 2D-ESPI.

**Figure A3:**
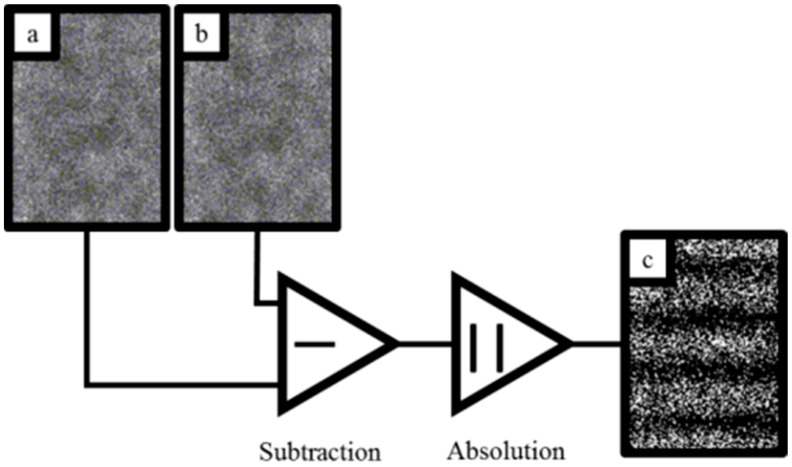
Speckle pattern of before deformation. (**b**) Speckle pattern of after deformation. (**c**) Fringe pattern image calculated from (**a**) and (**b**).

**Figure A4:**
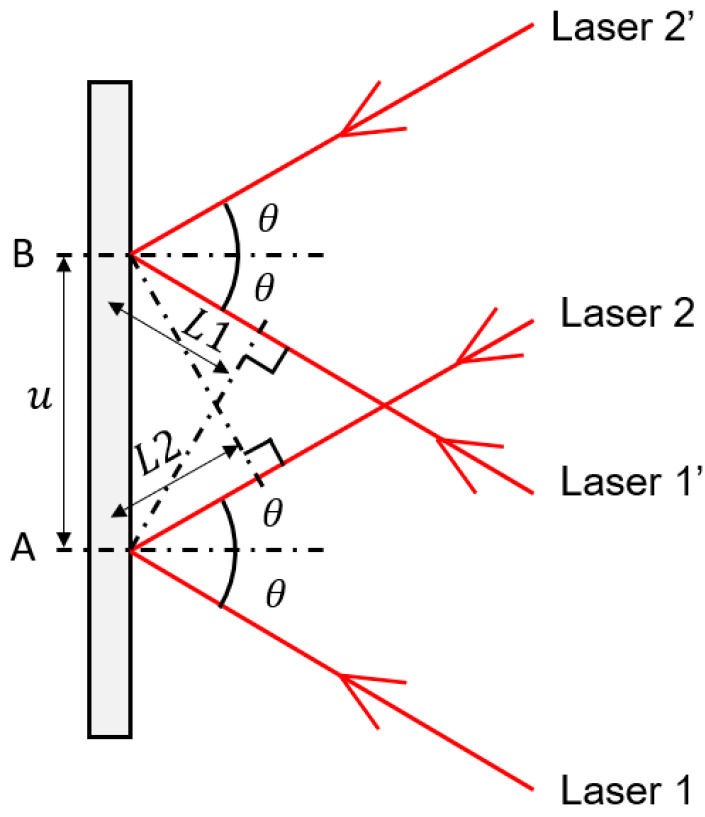
Schematic illustration of difference in optical path.

## Data Availability

The data presented in this study are available on request from the corresponding author.

## Appendix A. Principle of ESPI

ESPI is an optical measurement method that observes the displacement distribution on the measurement surface by photographing the speckle pattern that produced by the superposition of laser beams passing through two or more optical paths on the measurement surface with a Charge Coupled Device (CCD) camera and applying image processing. The displacement distribution obtained by ESPI is displayed as contour interference fringes, and the strain distribution can be obtained by analyzing the fringe pattern. ESPI advantages by its full-field of view, non-contact measurement, and large measurement area. In addition, ESPI can measure in real-time because the CCD camera and image processing can be performed simultaneously by a computer. ESPI can detect small displacements on the order of several hundred [nm] over a large measurement area.

The principle of ESPI is described below. When a laser beam illuminates the surface of a material, the laser beam is diffused reflected due to the roughness of the surface. Due to the interference of the diffused reflected laser, a speckled pattern is observed on the material surface (Figure A2). When the material is deformed, the interference conditions of the laser beam on the surface also change, and the intensity of the speckle pattern also change. Figure A2 shows a schematic diagram of the optical system for in-plane displacement measurement by ESPI. The laser beam is emitted from the semiconductor laser is expanded by the beam expander and then split into two directions by the beam splitter. The split laser beams are reflected by mirrors and illuminate from two directions symmetrical to the normal of the measurement surface. At this time, the two lights interfere each and form a speckle pattern on the specimen surface. The interference speckle pattern is recorded by a CCD camera, and the absolute value of the difference in intensity between the image before deformation (Figure A3a) and the image after deformation (Figure A3b) yields the stripe image as Figure A3c.

Figure A4 shows the optical path difference when the measurement surface illuminated by the laser beam moves u in the in-plane direction. The figure shows the case where two laser beams, Laser1 and Laser2, split by a half-mirror, are incident on point A and the surface moves u in the in-plane direction from point A to point B due to deformation of the material. In this case, assuming that Laser1’ and Laser2’ are incident on point B, the optical path difference between Laser1 and Laser1’ is $L_{1}=U\sin\theta$, and that between Laser2 and Laser2’ is $L_{2}=U\sin\theta$, and the optical path difference between the two directions is L=Usinθ. Next, we consider the change in the intensity of the laser beam when point A is displaced to point B due to the deformation of the material. The state equation E of the laser beam moving in the forward direction on the *x*-axis at time t(s) is expressed as follows:

(A1) $$E(x,t)=A\cos\frac{2\pi }{\lambda }(x-c_{a}t)=A\cos(kx-\omega t)$$

where *A* is the amplitude of the laser beam, *λ* is the wavelength, $c_{a}$ is the speed of light in the atmosphere, $k=\frac{2\pi }{\lambda }$, and $\omega =kc_{a}$. Expressing this using the complex function is as follows:

(A2) $$E(x,t)=Ae^{i(kx-\omega t)}$$

It follows that the intensity of the light at a fixed observation time *T(s)* is expressed as follows:

(A3) $$I(x,t)=\frac{1}{T}\int_{0}^{T}E\cdot E^{*}dt$$

Substituting Equation (A2) into Equation (A3)

(A4) $$I(x)=\frac{1}{T}\int_{0}^{T}Ae^{i(kx-\omega t)}\cdot Ae^{-i(kx-\omega t)}dt=A^{2}$$

The square of the amplitude of the laser light is the intensity of the light. From Equation (A2), the two laser beams irradiated to point A, Laser1 and laser2, can be expressed as follows:

(A5) $$U_{1}=A_{1}e^{i(kx-\omega t)}$$

(A6) $$U_{2}=A_{2}e^{i(k(x+\Delta x)-\omega t)}$$

$A_{1}$ and $A_{2}$ are the amplitudes of the respective laser beams, and Δx is the small optical path difference between Laser1 and Laser2 before the material deformation. This optical path difference generates an interference speckle pattern. The equation of laser beam at point A is expressed as a superposition of $U_{1}$ and $U_{2}$ as follows:

(A7) $$E_{A}=U_{1}+U_{2}=A_{1}e^{i(kx-\omega t)}+A_{2}e^{i(k(x+\Delta x)-\omega t)}$$

The intensity of the superposition of the two laser beams is as follows:

(A8) $$I_{A}=\frac{1}{T}\int_{0}^{T}\left(A_{1}e^{i(kx-\omega t)}+A_{2}e^{i(k(x+\Delta x)-\omega t)}\right)\left(A_{1}e^{-i(kx-\omega t)}+A_{2}e^{-i(k(x+\Delta x)-\omega t)}\right)dt\;=A_{1}{}^{2}+A_{2}{}^{2}+2A_{1}A_{2}\cos(k\Delta x)$$

The intensity of the interference speckle pattern of the two laser beams varies with kΔx. When point A is displaced to point B due to deformation of the material, the laser beams at point B, Laser1’ and Laser2’ express as follows:

(A9) $$U_{1^{\prime }}=A_{1}e^{i(k(x+L_{1})-\omega t)}$$

(A10) $$U_{2^{\prime }}=A_{2}e^{i(k(x+\Delta x+L_{2})-\omega t)}$$

The equation of the laser beam at point B and the Intensity of it are expressed as follows:

(A11) $$E_{B}=U_{1^{\prime }}+U_{2^{\prime }}=A_{1}e^{i(k(x+L_{1})-\omega t)}+A_{2}e^{i(k(x+\Delta x+L_{2})-\omega t)}$$

(A12) $$I_{B}=\frac{1}{T}\int_{0}^{T}\left(A_{1}e^{i(k(x+L_{1})-\omega t)}+A_{2}e^{i(k(x+\Delta x+L_{2})-\omega t)}\right)\left(A_{1}e^{-i(k(x+L_{1})-\omega t)}+A_{2}e^{-i(k(x+\Delta x+L_{2})-\omega t)}\right)dt\;=A_{1}{}^{2}+A_{2}{}^{2}+2A_{1}A_{2}\cos k(\Delta x+L_{1}+L_{2})\;=A_{1}{}^{2}+A_{2}{}^{2}+2A_{1}A_{2}\cos(k\Delta x+2ku\sin\theta )$$

The absolute value of the intensity of the laser beam before and after the deformation is as follows:

(A13) $$I_{A}-I_{B}=2A_{1}A_{2}\left\{\cos k\Delta x-\cos(k\Delta x+2ku\sin\theta )\right\}\;=4A_{1}A_{2}\sin(ku\sin\theta )\sin(k\Delta x+ku\sin\theta )$$

From this equation, the intensity of the interference speckle pattern due to deformation changes with kusinθ. Expressing this equation using the phase change as Δφ and k=2π/λ is as follows:

(A14) $$\Delta \varphi =\frac{4\pi }{\lambda }\sin\theta$$

Whenever the phase ∆*φ* becomes a multiple of 2*π*, the intensity of the interference speckle pattern becomes 0. Therefore, the fringe pattern (interference fringe) appears on a surface with a displacement gradient and the displacement when n interference fringes appear is expressed as follows:

(A15) $$u=\frac{n\lambda }{2\sin\theta }$$
